# Donepezil-induced psychosis: a cautionary report of a rare adverse reaction

**DOI:** 10.1192/j.eurpsy.2023.778

**Published:** 2023-07-19

**Authors:** K. R. Hutchings, B. K. Hutchings, B. Ratnakaran, A. S. Kablinger

**Affiliations:** 1 Virginia Tech Carilion School of Medicine, Roanoke; 2 Southern Utah University, Cedar City; 3 Carilion Clinic, Roanoke, United States

## Abstract

**Introduction:**

Donepezil is an acetylcholinesterase inhibitor approved by the Food and Drug Administration for the treatment of dementia in Alzheimer’s disease. While it is not curative for Alzheimer’s disease, donepezil has been shown to improve symptoms and slow disease progression; however, cases of rare psychiatric adverse effects, including hallucinations, mania, and increased confusion, have been reported. This report presents a case of donepezil-induced psychosis, which quickly resolved following cessation of the offending medication.

**Objectives:**

To illustrate a unique case of donepezil-induced psychosis

**Methods:**

The patient is an 81-year-old male with a history of late-onset Alzheimer’s disease, mild depression, hypertension, hyperlipidemia, gastroesophageal reflux disease, and myocardial infarction. The patient was prescribed oral donepezil 10mg twice daily to manage his late-onset Alzheimer’s disease. Subsequently, he began developing persecutory delusions, increased agitation toward his family, and auditory hallucinations. His symptoms were worse at night after taking the donepezil, and he regularly requested to have a firearm for self-defense in the late hours of the night. His symptoms progressed for several weeks before his family brought him into the geriatric psychiatry clinic to address his psychosis. The family recognized that these new symptoms started shortly after the patient began taking donepezil and had already started decreasing the dose to half of what was originally prescribed.

**Results:**

This patient experienced symptom remission from psychosis immediately upon discontinuation of donepezil. The patient and his family reported significant improvement with no continuation of hallucinations or paranoia. There was also reported improvement in mood and irritability, and the patient appeared significantly better upon follow-up with geriatric psychiatry. Due to this immediate improvement, the suspected causative factor in the precipitation of psychosis in this patient is the anticholinesterase activity of the donepezil. Although the prescribing information of donepezil details inadequate data proving an association between donepezil and psychotic symptoms, two other published case reports (Yorston GA *et al*. J Psychopharmacol 2000;14:303-4, Pozzi FE *et al.* Case Rep Neurol 2022; 14:359-365), along with this one, provide evidence of a causal relationship between the two. The patient was switched to memantine therapy and has remained free of psychotic symptoms thus far.

**Conclusions:**

This case demonstrates the caution required among clinicians when prescribing donepezil for the treatment of Alzheimer’s disease. There needs to be a more focused risk evaluation of potential psychiatric adverse effects in patients treated with donepezil.

**Disclosure of Interest:**

None Declared

